# Surgical management of moderate adolescent idiopathic scoliosis with ApiFix®: a short peri- apical fixation followed by post-operative curve reduction with exercises

**DOI:** 10.1186/s13013-015-0028-9

**Published:** 2015-02-05

**Authors:** Yizhar Floman, Gheorghe Burnei, Stefan Gavriliu, Yoram Anekstein, Sergiu Straticiuc, Miklos Tunyogi-Csapo, Yigal Mirovsky, Daniel Zarzycki, Tomasz Potaczek, Uri Arnin

**Affiliations:** Israel Spine Center at Assuta Hospital, Tel Aviv, Israel; MS Curie Children’s Hospital, Bucharest, Romania; Spine Unit Assaf Harofeh Hospital, Zrifin, Israel; St. Maria Hospital, Iasi, Romania; Pecsi Orthopediai Klinika, Pecs, Hungary; Orthopedic Surgery, University Hospital Zakopane, Zakopane, Poland; ApiFix, Misgav, Israel

**Keywords:** Moderate AIS, Main thoracic curve, Short fixation, Correction with exercises

## Abstract

Surgery in adolescent idiopathic scoliosis (AIS) is a major operative intervention where 10–12 vertebrae are instrumented and fused. A smaller motion preserving surgery would be more desirable for these otherwise healthy adolescents. The ApiFix® system is a novel less invasive short segment pedicle screw based instrumentation inserted around the apex of the main curve. The system has a ratchet mechanism that enables gradual postoperative device elongation and curve correction. The ratchet is activated by performing specific spinal exercises. The unique features of the device allow curve correction without fusion. The system which has a CE approval was employed in adolescents with main thoracic curves.

More than a dozen of ApiFix surgeries have been performed so far. The preoperative Cobb angle was 45° ± 8, and 25° ± 8 at final follow up. The following is a report on three adolescent females aged 13–16 years with curves between 43°-53° and Risser sign of 1–4 who underwent surgery with ApiFix®. Two pedicle screws were inserted around the curve apex and the ratchet based device with polyaxial ring connectors was attached to the screws. No fusion attempt was made. Operative time was around one hour. Two weeks after surgery the patients were instructed to perform Schroth like daily exercises with the aim of rod elongation and gradual curve correction. Patients were followed between 6 months to 2 years. Curves were reduced and maintained between 22- 33°. Patients were pain free and were able to perform their spinal exercises. Postoperative gradual elongation of the device was observed. No screw loosening or rod breakage were observed. No adding on or curve progression was seen.

Three factors may contribute to the ApiFix® success: polyaxial connections that prevent mechanical failure, gradual curve correction by spinal motion and spinal growth modulation. The ApiFix® system allows managing moderate AIS with a simple and minor surgical intervention. Recovery is rapid with negligible motion loss. It allows gradual and safe curve correction with high patient satisfaction. It may also serve as an internal brace for AIS.

Adolescent idiopathic scoliosis (AIS) is a condition that affects 1%-3% of children aged 10–16 [1]. It is a structural lateral curvature of the spine with a significant rotatory component that develops in healthy teenagers around the puberty period. In its mild or moderate forms AIS pauses no health threats but may be associated with cosmetic concerns. As the name idiopathic implies, no known cause has been identified and therefore no specific treatment exists. Individuals with curves up to 20-25° are advised to exercise usually according to the Schroth method. Curves between 25-40° are braced with some kind of a thoraco-lumbar spinal orthosis. Patients with curves exceeding 40-45° are candidates for surgical intervention [1], the “gold standard” being instrumentation and fusion of 10–12 vertebrae with intra-operative forceful correction of the spinal deformity. This “gold standard” of curve correction is an extensive and irreversible surgical endeavor that stiffens the spine of adolescents who are otherwise healthy youngsters. Today’s continuum of care of AIS has a steep leap from non operative care to the most extensive and complex spine surgery. This complex “gold standard” surgery is applied to different curve patterns and to various curve magnitudes in an almost identical fashion. It is obvious that we lack an intermediate surgical step that will address mild to moderate AIS curves with a less invasive and less extensive surgery, yet will provide efficient correction and control of the deformity. How to set the bar of success for such intermediate type surgery? It may be borrowed from the bracing studies where a maximum of 40-45° residual deformity is considered a success [2,3].

The ApiFix device has been developed to address this missing step and to provide an effective method of controlling the deformity. The device is implanted in a less invasive fashion and with fewer instrumented segments.

The ApiFix® system is a novel less invasive short segment pedicle screw based spinal instrumentation (3–4 disc levels). The system has a ratchet mechanism that enables gradual postoperative device elongation and curve correction by spinal exercises (Figure 1a). The unique features of the device allow spinal instrumentation without fusion. Currently the device may be implanted in AIS patients with main thoracic or thoraco-lumbar curves, before the end of skeletal maturation.

**Figure 1:**
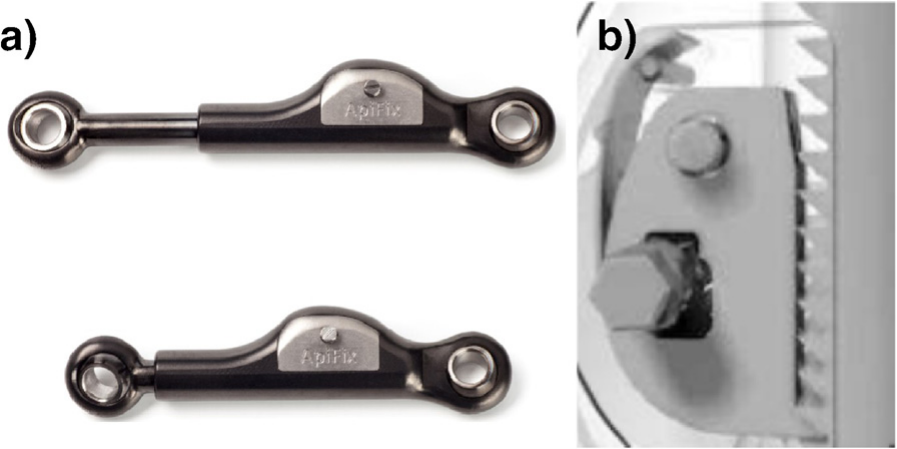
**The ApiFix short peri-apical scoliosis correction device. a**: The ApiFix device. **b**: The mini ratchet mechanism.

The implanted ApiFix acts as an “internal brace”. Following implantation of the ApiFix system the initiation of early post operative exercises gradually corrects the spinal deformity by motion controlled rod elongation.

## Implant design

The device has an overall length range of 65–105 mm, and is expanded in increments of 1.3 mm, for total extension of 20–30 mm, depending on length. The device has a mini-ratchet mechanism that allows unidirectional elongation of an expandable rod. The mini ratchet mechanism consist of a toothed area and locking tooth (Figure 1b). Both are made from Titanium alloy. The locking tooth rotates around a 2 mm pin and interacts with the toothed area to allow only unidirectional movement. The locking tooth is pressed via a flat spring to prevent backwards slippage. The spring part is made from Nitinol (NiTi). The entire device is made from Titanium alloy with ADLC coating (amorphous diamond-like ceramic). This ceramic coating minimizes friction and wear. The ceramic coating has also the ability to inhibit bacterial growth that may reduce the incidence of post operative deep wound infection. The expandable rod with polyaxial rings at its extremities, is attached by 2 pedicle screws around the apex of the main spinal curvature. As stated before the rod can expand by either 20 mm or 30 mm depending on the pre-distraction rod length. Rod expansion is incremental and gradual, making the deformity correction safer than “all at once” acute rod distraction in the standard type surgery. Spanning the correction process over several weeks or months allows the soft tissues to accommodate any incremental correction. The long, incremental process reduces the load on the screws and allows the body to slowly rebuild itself in the correct position/shape.

The implant has a control pin that can abort the ratchet mechanism and put the device in a free neutral mode or a locked position creating a stiff fusion like rod.

### Surgical technique and postoperative exercises

The concave side of the spine is exposed through a 10 cm incision around the curve apex. The contra-lateral side is left undisturbed. Two peri apical pedicular screws, spanning 3 or 4 motion segments are inserted. The ApiFix system is connected to the pedicle screws. Initial distraction during surgery allows some correction of the deformity. No fusion is performed. The surgical procedure takes about an hour and blood loss is negligible. Intraoperative neuro-monitoring or a “wake-up” test may be used. Two to three weeks after the surgery the patients are directed to perform five basic Schroth exercises that enable gradual elongation of the ratchet mechanism leading to reduction of the spinal curve. These exercises consist of hand hanging from a bar or door, lateral bending maneuvers while standing or sitting and side stretching while lying on the side over a firm roll. The patient is instructed to perform the exercises for 30 minutes on a daily basis. Exercises are continued for 3–6 months after surgery. The device is CE approved.

### Illustrative case report

#### Case report 1

**Figure 2:**
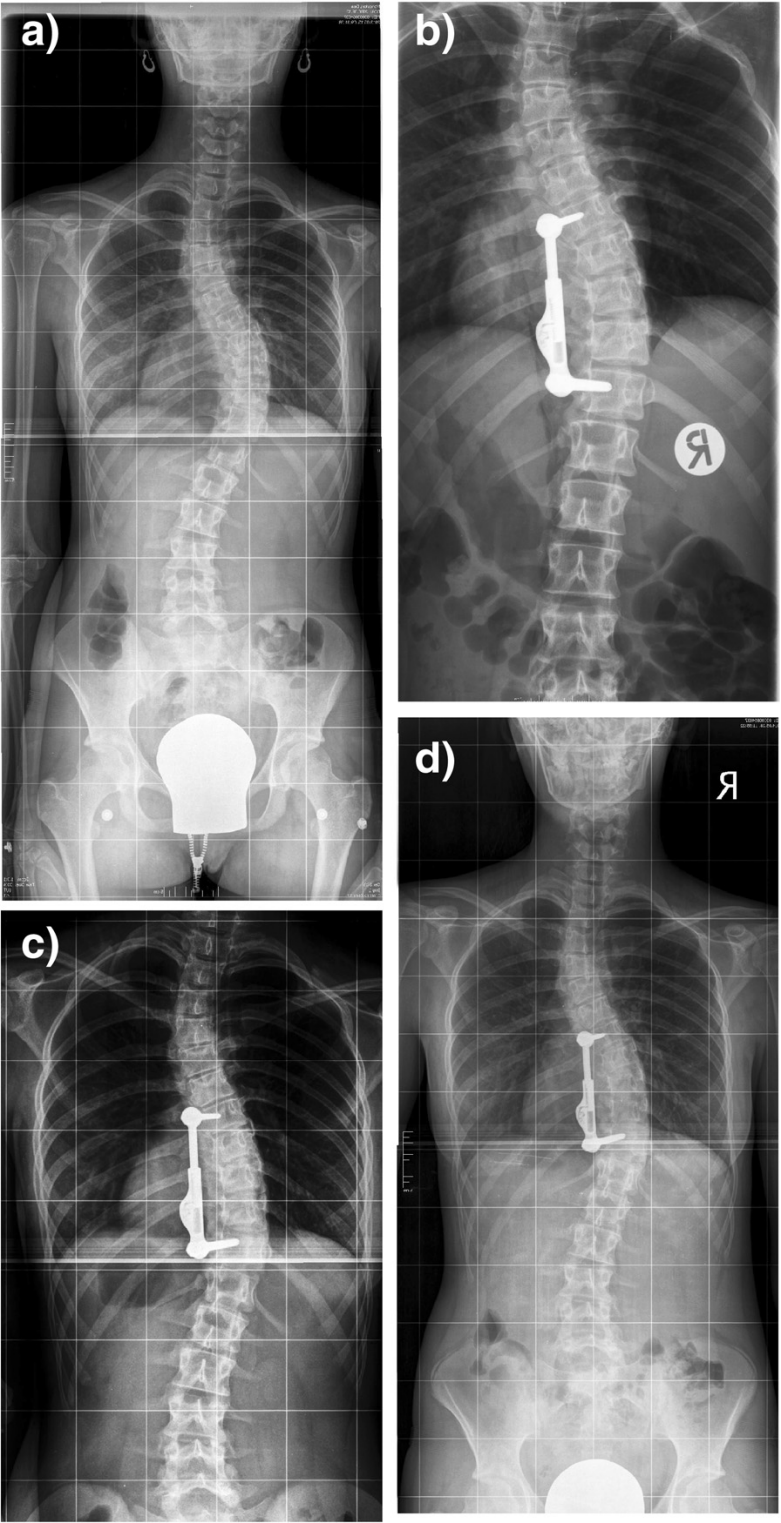
**13 year old girl with right thoracic scoliosis. a**: The preoperative Cobb angle is 53°. **b**: postop. x ray after instrumentation T7-11 and initiation of exercises. **c**: elongation of the ApiFix with exercises. **d**: at one year follow up 33°.

A 13 year old female presented with a 53° main thoracic curve, the Risser sign was 1. Lateral bending x rays showed curve correction to about 50%. A T7-11 instrumentation was performed. The curve was gradually corrected to 33° (Figure 2) and maintained at this magnitude at 1 year follow up. The instrumentation was found to be intact with no evidence of screw loosening. The patient is pain free and satisfied with her appearance.

#### Case report 2

**Figure 3:**
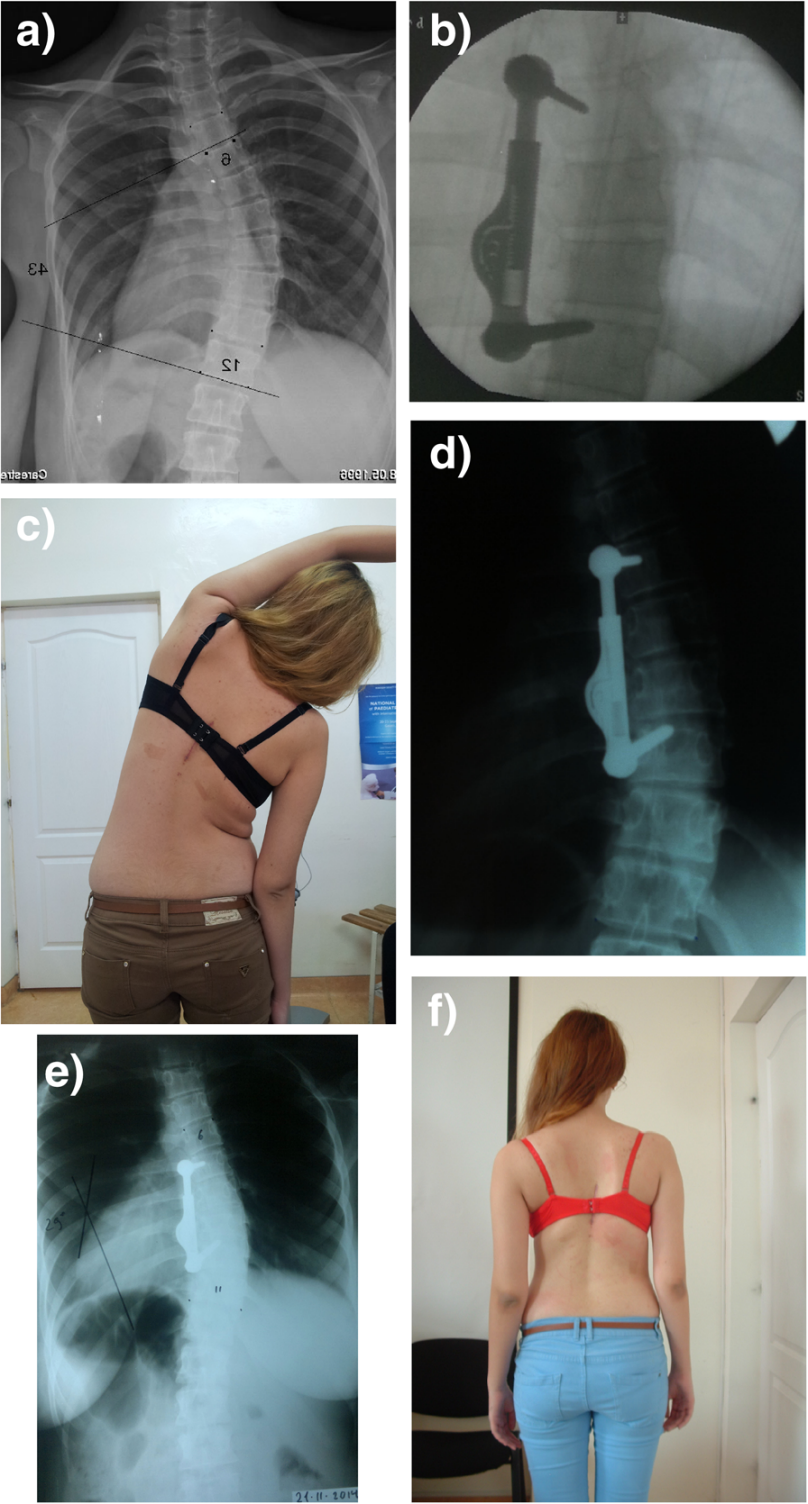
**16 year old girl with right thoracic scoliosis. a**: The preoperative Cobb angle is 43°. **b**: after instrumentation from T7-10. **c**: Patient is exercising 6 weeks after surgery. **d**: device elongation with exercises. **e**: Follow up x ray at 2 years. 25° curve. **f**: clinical appearance of patient.

A 16 year old female presented with a 43° main thoracic curve, the Risser sign was 3. The curve was supple on side bending x rays and showed a significant curve diminution. She was operated and instrumented from T7-10. The curve was gradually corrected to 29° with exercises (Figure 3b) and finally stabilized at 25° at two years follow up (Figure 3e). No evidence of implant related adverse events was present. Her cosmetic appearance was much improved (Figure 3f)

#### Case report 3

**Figure 4:**
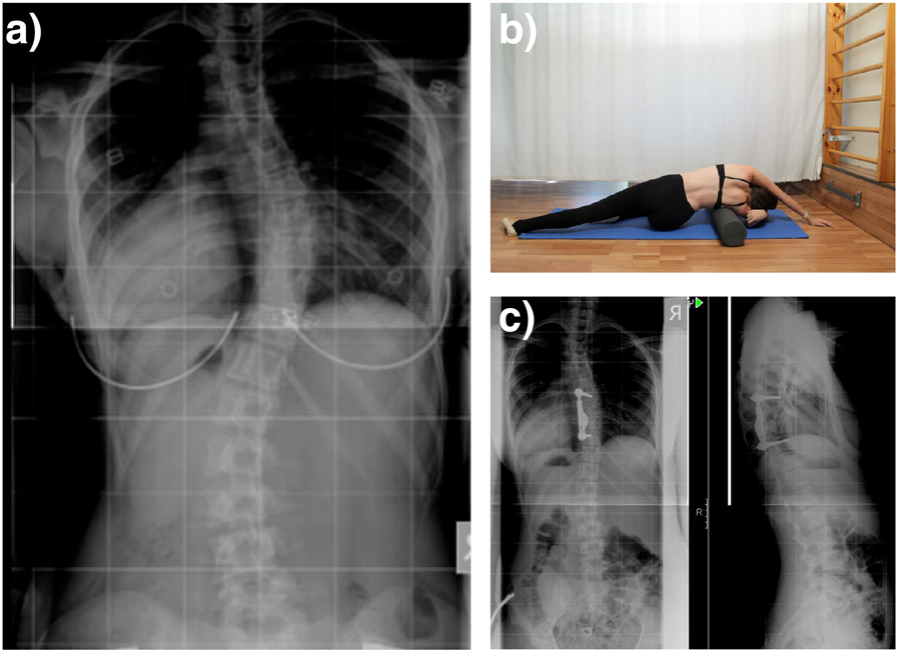
**15 year old girl with right thoracic scoliosis. a**: The preoperative Cobb angle is 45°. **b**: postoperative exercises. **c**: curve correction to 22° at 6 month follow up.

A 15 year old female presented with a 45° main thoracic curve (Figure 4). Risser sign was 4 and curve corrected to 20° on side bending x rays. After implantation of the ApiFix (T7-T10) and postoperative exercises the curve was corrected to 22 at 6 months follow up (Figure 4). The patient is well balanced and the implants are well secured to the spine.

## Discussion

There is a clear need for fusion-less scoliosis correction performed in a less invasive fashion. Following surgery a rapid recovery and a preservation of spinal motions are anticipated. The need for this new type surgery is shared by both patients and surgeons. In the current reported cases, the ApiFix system proved successful in reaching both goals.

So far more than a dozen of ApiFix surgeries have been performed. Surgery was short and uneventful and hospitalization was 2–3 days at most. After initial curve correction during surgery, further correction is achieved with postoperative exercises by activation of the mini-ratchet mechanism. In most cases curves were reduced to below 25-35° and maintained at these levels at follow up visits ranging from 6 months to 2 years. No screw loosening or breakage were encountered. Patients are pain free with minor restriction of back motion.

Surgeons confronted with such case reports managed by a novel non conventional surgical treatment usually raise several concerns and doubts. Would secondary curves be corrected, would curves above or below the implant develop and progress? They would also state that in the absence of fusion, there is a high probability of implant failure with screw breakage or loosening. These are certainly legitimate concerns. However a closer look at the literature regarding the management of main thoracic curves will refute the concerns regarding the fate of the secondary curves and close examination of the mechanical design of the ApiFix can refute the grim scenario of implant failure.

Lenke et al. [4] in a mid-term follow up and Larson et al. [5] in a long term follow up of selective instrumentation and fusion of main thoracic curves in AIS showed significant reduction of the secondary curves and maintenance of the correction as long as 20 years after surgery (4.5). Biomechanical test on the run out load of 5000000 cycles showed the ApiFix to be 3 fold stronger than standard fusion constructs (unpublished data). This can be explained on the basis of the unique spherical joint between the implant and the screw (Figure 1). This poly-axial connection prevents mechanical moments to be transferred to the screw except in pure axial compression. This explains why a stiff fusion construct will fail whenever bony fusion has failed to occur while the ApiFix construct will remain intact even without concomitant fusion.

Short apical fixation without fusion defies conventional wisdom of scoliosis correction. Three factors may contribute to the ApiFix® success: polyaxial connections that prevent mechanical failure, gradual curve correction by spinal motion and spinal growth modulation. The ApiFix® system allows managing moderate AIS with a simple and minor surgical intervention. Recovery is rapid with negligible motion loss. It allows gradual and safe curve correction with high patient satisfaction. It may also serve as an internal brace for AIS.

The current indications for ApiFix surgery in AIS are as follows: 11–16 years teenagers with main thoracic or thoracolumbar curves between 40-60° correcting by 40-60% on side bending films. The percentage of correction of the deformity with the ApiFix is somewhat smaller as compared to the gold standard posterior surgery [6]. Does it really matter if the curve was reduced to 35° 25° or 15°? Several studies have shown that if the Cobb angle is reduced to 35° or less the SRS-22 outcome score will be the same for curves corrected to 35° or 25° or 15° [7,8].

The ApiFix surgery is a simple and short procedure especially when compared to the “gold standard” long fusion. Common sense would also indicate that it is associated with much lower adverse events and complication than the usual standard surgery. Surgery takes less than an hour as compared to 4–5 hours with long fusion (Table 1). Blood loss is negligible, no neurologic complications have been encountered so far (Table 1). The safe neurological outcome can easily explained by the reduced number of vertebrae instrumented with pedicle screws and the gradual smooth correction of the deformity with the ApiFix way (between 2 weeks to a couple of months). Smorgick et al. found that placement of pedicle screws at the concavity of the deformity and placement of screws above T8 were risk factors for screw malplacement and potential neural damage [9]. Since ApiFix requires only two concave screws to be inserted, none at the apex itself, and only one screw is placed above T8, the danger of screw related neurological deficit is markedly reduced.

**Table 1:** **Comparison between standard instrumentation and fusion and ApiFix instrumentation without fusion**

**Standard long fusion**	**ApiFix**
incision-30 cm	10 cm
Time-238 min.	60 min.
Blood loss-800-1500 cc	50 cc
% correction-57%	46%
Neuro. comp.-0.8%	so far none
Non neuro. comp.-11.5%	?
Pseudoarthhrosis-4%	none
Revision surgery-18%	none
Back pain-30-40%	none ?

As no attempts at fusion are made no pseudoarthroses occur with ApiFix while in the standard method there is a 4% incidence of pseudoarthrosis [6]. Non neurologic complications prevalent with the standard methods are also rare with ApiFix [10]. Motion is far less restricted with ApiFix and it may be predicted that this will be expressed with less frequent complaints of future back pain and less occurrence of adjacent level disc degeneration.

Of special interest is the comparison of the standard surgical treatment of Lenke type 1 curves (main thoracic curve) to the same type of curve instrumented with the less invasive ApiFix procedure. Newton et al. [6] presented theirs, and other centers of excellence, experience with managing Lenke type 1 curves. This was a prospective multicenter study. Incision length was 28.5 cm, an average of 10 levels were instrumented, operative time was 238 minutes, and blood loss was 807 cc. The average preoperative Cobb angle was 49° and the percentage of deformity correction was 57% [6]. Implant failure was noted in 3% and reoperation rate was 2%. The rate of major complications was 8% and minor complications 28%. With ApiFix only 3–4 levels are instrumented, operative time is less than 60 minutes, incision length is 10 cm, blood loss 50 cc, no fusion is performed and the percentage of deformity correction is around 50%. No implant failure or unplanned return to the operating room were encountered. It looks as though that the use of ApiFix° in Lenke type 1 curves should be the preferred method in moderate Lenke type 1 curves.

With the use of ApiFix no bridges are burnt and in the “worst” scenario a standard posterior instrumentation and fusion can be still performed. It may be summarized that the evolution of AIS surgery has taken a direction toward limitation of the number of instrumented vertebrae by the operative construct and with a fusion less surgery [11]. ApiFix certainly represents well these goals.

In summary there are many drawbacks to the current gold standard of AIS surgery which are almost nonexistent with the use of ApiFix: considerable blood loss leading to blood transfusions [12], neurologic deficit including cord lesions, a 12% prevalence of a host of non neurologic complications [10], late infections, pseudoarthrosis, limitation of spinal motion also affecting non fused levels, high concentrations of metal ions in the blood, back pain and disc degeneration in the non fused spinal segments. Almost all complications can be avoided by the use of ApiFix, minimal blood loss with no need for blood transfusion, negligible prevalence of non neurologic complications, neurologic complication are theoretically avoided by the use of two pedicle screws and the gradual nature of the deformity correction, no pseudoarthrosis as fusion is not attempted, late infection may be eliminated due to the special antibacterial coating of the ApiFix implant, minimal restriction of spinal motion with minor or no effect on adjacent non instrumented levels. As with every novel spinal system, the key for success is proper indications and strict patient selection. Longer term clinical trials with a larger patient cohort are surely needed.

## Consent

Written informed consent was obtained from the parents or legal guardians of the patients for publication of this article and any accompanying images. A copy of the written consent is available for review by the Editor-in-Chief of this journal.
